# Social exclusion and psychopathology in an online cohort of Moroccan-Dutch migrants: Results of the MEDINA-study

**DOI:** 10.1371/journal.pone.0179827

**Published:** 2017-07-10

**Authors:** Madelien H. van de Beek, Lian van der Krieke, Robert A. Schoevers, Wim Veling

**Affiliations:** 1 Dimence Institute for Mental Health, Dimence Group, Zwolle, the Netherlands; 2 University Center for Psychiatry, University Medical Center Groningen, University of Groningen, Groningen, the Netherlands; Maastricht University, NETHERLANDS

## Abstract

**Introduction:**

Migration is seen as a risk factor for developing psychiatric symptoms and experiencing social exclusion. In the Netherlands, the Moroccan-Dutch population is the second largest migrant group. 70% of all young Moroccan-Dutch people meet each other in the online community www.marokko.nl. Within this community, we investigated the association between experiences of social exclusion and self-reported depressive symptoms and psychotic experiences.

**Materials and methods:**

Participants were recruited via the website www.marokko.nl. They completed an online survey, with screening instruments for depressive symptoms (K10) and psychotic experiences (PQ-16), measures of social exclusion (perceived discrimination, social defeat and social support), and questions about demographical information. With regression analysis the association between social exclusion and psychiatric symptoms was investigated.

**Results:**

We included 267 participants; 87% were female. 27% of the sample has received mental healthcare in the past. Over 50% of these people screened positive for depressive symptoms and psychotic experiences. Perceived discrimination and social defeat were significantly associated with psychotic experiences and social defeat was associated with depressive symptoms. Social support and higher education were associated with less depressive symptoms and psychotic experiences.

**Discussion:**

Our findings suggest that the online environment allows for epidemiological research and early symptom detection. Levels of psychopathology were high in our sample. This suggests that a part of this young ethnic minority population might not get adequate mental healthcare. Since this population can be reached through Internet, the online environment may therefore also offer an appropriate setting for intervention, to increase resilience towards social exclusion.

## Introduction

Across countries, migration is a risk factor for the development of a range of psychiatric disorders. Two meta-analyses have shown a strong association between migration and psychotic disorders [1,2]. A recent meta-analysis showed that the risk for non-affective psychosis and subclinical psychotic symptoms was increased in male, but not female, migrants from Morocco and surrounding countries [3]. An association between migration status and mood disorders was also found in a meta-analysis [4] and confirmed in several epidemiological studies [5,6]. In 2013, Cantor-Graae and Pederson showed an increased risk for the full spectrum of psychiatric disorders in migrants, with the second generation migrants having increased risk for more psychiatric disorders compared to the first generation [7]. Also in the Netherlands, migrants have increased risk for psychiatric disorders. The risk for schizophrenia spectrum disorders is increased in first and second generation migrants. [8,9] There is also evidence that the prevalence of depressive disorders is increased in Turkish-Dutch and Moroccan-Dutch people compared to native Dutch people [9,10]. The incidence of psychotic disorders is not only increased in first generation migrants, but also in second-generation migrants [1,2]. Furthermore, the age of migration has been found to be negatively correlated with the risk of developing a psychotic disorder.[11]. This suggests that (social) factors in the country of destination are important [12,13]. In other words: the ethnic minority status is more important than the actual migration itself [1,2,14].

Which factors make the ethnic minority status a risk factor for mental health problems? In the meta-analysis of Cantor-Graae *et al* (2005), ‘black skin colour’ was associated with a higher relative risk for psychosis compared to migrants with a non-black skin colour. This finding points towards an underlying mechanism of discrimination, since people with black skin colour are more often discriminated against [2]. Indeed, discrimination has previously been associated with psychotic symptoms and mood disorders [15–21]. Several other social concepts are associated with psychopathology. Social defeat, which is defined as ‘being in a subordinate position’, by Gilbert and Allan in 1998, is associated with depression [22–25] and has been associated with psychosis [26]. A review about older Asian migrants in the USA describes a link between the prevalence of depression and several social factors, including social support and acculturation [27].

Psychiatric disorders like psychosis and depression can be considered as a continuum in early stages of the disorder [28] and were shown to co-occur more frequently in Moroccan- and Turkish-Dutch migrants compared to native Dutch people [8]. Early detection of individuals with high risk of developing psychopathology is important, in order to deliver adequate preventive measures [29,30]. Early detection is mostly performed in persons who actively seek help [29]. This study is performed in a ‘general population’ online community of Moroccan-Dutch people. With this study, we can test the feasibility of early detection within this population. Furthermore, the main results might give clues for developing preventive measures.

The Moroccan-Dutch population is the second largest migrant population in the Netherlands (380,755 inhabitants, reflecting 2,3% of the total Dutch population) [31]. Since this Moroccan-Dutch population is at increased risk for psychiatric disorders, we would like to identify high-risk individuals within this subgroup of the general population. Although this population is geographically spread across the Netherlands, they meet each other online. The majority (70%) of young Moroccan-Dutch people actively visit the website Marokko.nl, which is a news site and discussion forum, where they exchange information and opinions on many different subjects. This creates the opportunity to reach this population via the Internet for research purposes [32]. In this online community visitors are protected by anonymity. Interestingly, the website contains many forum discussions about psychiatric problems, a subject which is otherwise taboo in this population. This platform therefore creates an opportunity to investigate mental health problems in a high-risk ethnic minority population outside of the conventional mental healthcare setting.

### Aim of the study

In the MEDINA (Migrants Examined for Determinants of psychopathology through INternet Assessment) study, we used the online platform www.marokko.nl to investigate the association between symptoms of psychopathology (depressive symptoms and psychotic experiences) and experiences of social exclusion (reported measures of social defeat, perceived discrimination and social support) in a cross-sectional sample of young Moroccan-Dutch participants.

## Material and methods

We performed an online survey in a cross-sectional convenience sample of the Moroccan-Dutch population, using a combination of several self-report questionnaires. The research protocol of this study is described in a design paper [32]. We will here summarize the methods.

### Ethics

The protocol was presented to the Medical Ethical Committee (METc) of the University Medical Center Groningen (UMCG) and they decided that, due to the very limited burden and risks for participants, it was exempted from further review (reference number M12.124959). We obtained informed consent from participants (see further). Data were anonymously obtained via internet. IP addresses were not recorded. Privacy sensitive information (email addresses) was anonymised before analysis.

### Participants

Inclusion criteria were: Dutch citizenship; participant is born in Morocco or at least one of the parents is born in Morocco; having sufficient knowledge of the Dutch language and being a visitor to the website Marokko.nl. Exclusion criteria were: age younger than 18 years.

### Recruitment and consent

Marokko.nl is a very popular website, which is regularly visited by 70% of all young Moroccan-Dutch people. We invited participants via advertisement on the website Marokko.nl, shown as banners. For these advertisements, we used two different approaches.

In the first approach, we invited people to fill out the K10 as a depression self-test. As this measurement is part of the survey, these questions could be skipped at a later stage. The advertisement text was: ‘are you feeling down? Fill out the test!’. In the second approach, we asked people to participate in scientific research. The advertisement text for the second approach was: ‘Do you want to participate in scientific research?’. For the third recruitment strategy, we approached visitors of the website marokko.ziekofbezeten.nl (translation: being ill or being possessed). This is an add-on website of Marokko.nl, where information about psychiatric disorders and related religious phenomena is given and healthcare workers can be contacted. On this website, we used an opt-in method to ask if people agreed to be contacted on a later moment for scientific research. The people who agreed were invited for the MEDINA-study with an email invitation. We recorded for all participants via which approach they were recruited for the survey. We raffled gift vouchers between participants as an incentive.

At the first webpage of the survey, information about the study was presented, including the link to a file with complete participant information. At the same webpage, participants had to give informed consent, using a checkbox. After consent was given, people had to declare that their age was above 18 years old, before they could proceed to the survey.

### Measurements

The survey combines several, previously validated, short questionnaires, presented to the participants as a single online survey, which takes about ten minutes to complete. We used the Kessler Psychological Distress Scale 10 (K10), which has originally been designed for measuring distress, but has been validated within the Moroccan-Dutch population as a screening instrument for detecting clinically relevant depressive symptoms [33–35]. We used the Prodromal Questionnaire-16 (PQ-16) for screening for psychotic experiences [36]. The PQ-16 included an impact scale, that assesses the distress of the experience. For each item this distress was scored between 1 (no impact) and 4 (high impact). We measured perceived discrimination with the Every Day Discrimination Scale, which contains questions about recent experiences of personal discrimination [37–40]. Social support was measured with the ‘Oslo Social Support Questionnaire’ [41] and social defeat was measured with the ‘Social Defeat Scale’ [22]. Furthermore, we included socio-demographic items assessing age, gender, first/second generation migrant status, previous mental healthcare and highest completed level of education. All demographic items were dichotomised. Age was based on median value (23 years). Education was split in ‘low’ (no diploma, primary school, (preparation) lower and intermediate vocational education) and ‘high’ (secondary education, higher vocational education, university). After filling out the online survey, participants received feedback about their personal scores on the psychopathology screeners.

### Additional questionnaires

At the end of the survey, we asked participants if they were interested in filling out two additional questionnaires. The first was social comparison, measured by the Social Comparison Scale [42]. This scale reflects participants’ self-image, compared to native Dutch people. Lower scores correspond with feeling inferior to others. The second questionnaire is the Acculturation Scale [43]. This scale is based on Berry’s model, which measures two dimensions of acculturation: to which extent people are oriented towards the Dutch society and to what extent towards the Moroccan society. With this scale, four acculturation style can be determined: Integration (high orientation towards Dutch society, high orientation towards Moroccan society); Segregation (low orientation towards Dutch society, high orientation towards Moroccan society); Marginalization (low orientation towards Dutch society, low orientation towards Moroccan society); Assimilation (high orientation towards Dutch society, low orientation towards Moroccan society) [44]. Grouping of individuals into different acculturation styles was based on median scores within our sample [45,46].

### Statistical analyses

Levels of depressive symptoms and psychotic experiences were measured on continuous scales with the K10 and the PQ-16 respectively. Proportions of participants with clinically relevant symptoms were calculated using cut-off scores as described in the literature. For the paper-and-pencil K10, the best cut-off was 22,5 for Moroccan and Turkish participants, compared to 16,5 for Dutch participants [33]. In an online Dutch population, the cut-off scores with the best sensitivity and specificity were 29, 31 and 32 [45]. We selected the most conservative cut-off score that was recommended in the online sample, which is 32 [45]. The only available cut-off score for the PQ-16 is 6, which is based on an offline sample of young adults seeking help for nonpsychotic disorders [35]. There is no other information about this instrument in an online or migrant populations.

We created sum scores for the social exclusion variables: social defeat, perceived discrimination and social support. We used Pearson correlation to evaluate the association between depressive symptoms and psychotic experiences.

For the main outcomes, we used regression analyses, consisting of three steps: In step one, we performed separate linear regression analyses for the two main outcomes: depressive symptoms (K10 sum score, continuous outcome measure) and psychotic experiences (PQ-16 sum score, continuous outcome measure). Both main outcomes were investigated in separate analyses with the variables social defeat, perceived discrimination, and social support to examine their unique contribution. These factors of social exclusion are overlapping concepts (Pearson correlation: social support and social defeat r = -0.470; social support and discrimination r = -0.312; discrimination and social defeat r = 0.287) and were therefore investigated separately. In step two, we performed multivariate linear regression, extending the previously described models with demographic parameters, using the ‘Enter’ method. The demographic parameters added were: gender (male/female) age (low <23 / high ≥ 23), migrant status (first/second generation), education (low/high). In step three, the variable social support was added to the analyses of perceived discrimination and social defeat, to investigate the hypothesized beneficial effect for social support on the outcome. We assessed whether the beta coefficient for social support was statistically significant. and evaluated whether the beta coefficients of the other social exclusion variables changed. If so, we added an interaction term to the regression model of the social exclusion variables and social support. All analyses were performed using SPSS version 20.0 (SPSS Inc., Chicago).

### Additional analyses

A subset of the participants completed additional questionnaires on social comparison and acculturation. For social comparison, the subsequent data analysis was similar as what had been done for the other variables: step one: linear regression with the dependent variables depressive symptoms and psychotic experiences; step two: multivariate linear regression including demographic parameters, and step three: adding social support. For acculturation, we created three dummy variables, based on the acculturation styles: ‘separation’, ‘assimilation’ and ‘marginalisation’. The acculturation style ‘integration’ was used as comparison. We thus examined the difference in psychopathology between integration and the three other acculturation styles. For the regression analysis, the three steps were followed, as described above.

### Sensitivity analysis

Within this cross-sectional design, by definition, we do not have information about temporality of the investigated associations. We hypothesize that social exclusion is a risk factor for developing psychiatric disorders. An association in the opposite direction is a possible bias of reversed causality. We do however have information about whether participants received previous mental healthcare. When social exclusion would be the consequence of having a psychiatric disorder, it can be expected that experiences of social exclusion are higher and associations between social exclusion and symptoms are stronger within the group with previous mental health complaints compared to the group of treatment-naïve participants. To explore this possible bias, we compared the levels of social exclusion between these groups. Furthermore, we performed regression analyses for the participants with and without a history of mental healthcare, using split file analyses. We assessed whether the explained variance of the model would be higher in the group with previous treatment, compared with the treatment-naive group. Furthermore, we compared whether the strength of the associations would change in the split file analyses, comparing the beta coefficients for the total sample with the beta coefficients for the treatment-naïve group.

## Results

### Sample characteristics

**Fig 1:**
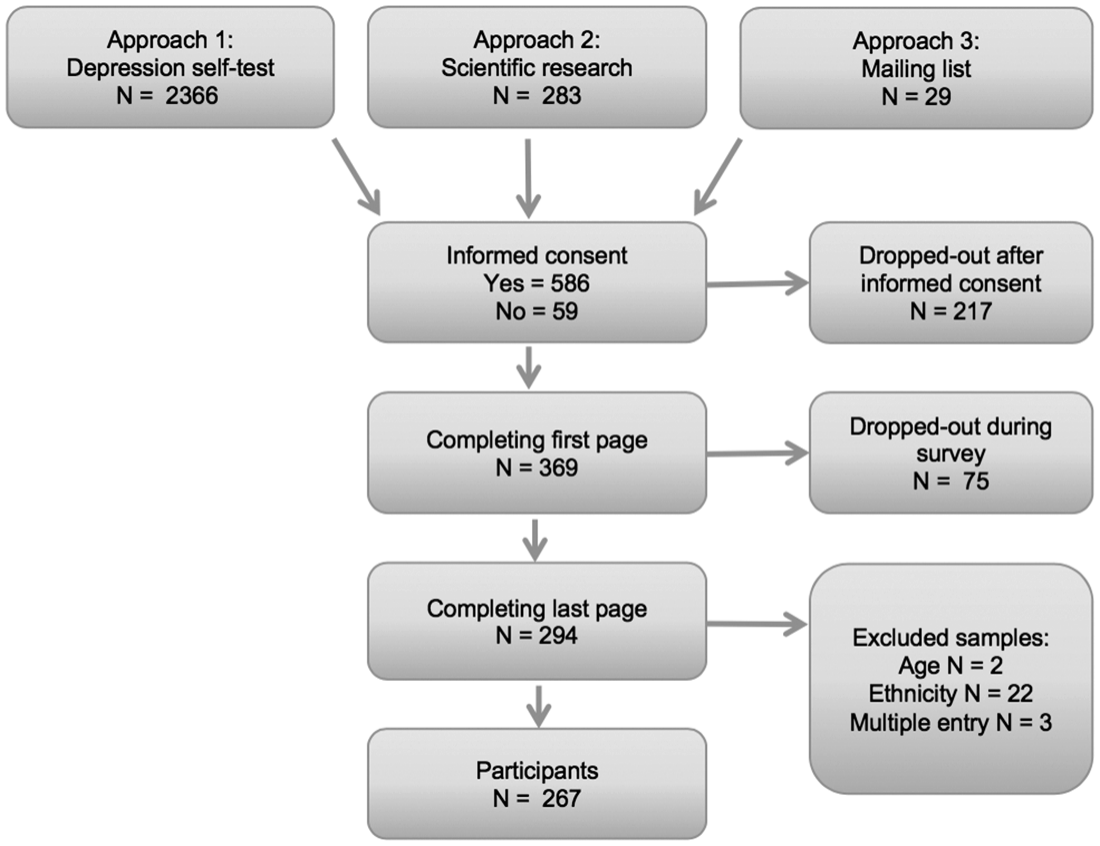
Flow-chart participants.

A total of 267 participants were included in the study between November 2012 and august 2014, see flow-chart in Fig 1. 87% (N = 231) of the participants were female. 81% were second generation Moroccan migrant, meaning they were born in the Netherlands and one or both of the participants’ parents were born in Morocco. The remaining 19% were first generation migrant. The mean age of the participants was 24,5 years (range 18–57). 27% percent of the sample received mental healthcare in the past. Demographic variables are shown in Table 1.

**Table 1:** Demographic information, psychiatric symptoms and measures of social exclusion.

**Demographic information**		**Number**	**Percentage**
Female gender		226	86.6%
Second generation migrant		211	80.8%
Previous mental healthcare treatment		69	27.2%
Higher education		76	29.5%
	**Median**	**Mean**	**Std. Dev.**
Age (range 18–57)	23	24,5	6,7
**Symptoms**			
Depressive symptoms (range 10–50)	33	32	9,6
Psychotic experiences (range 0–16)	6	6	4
**Social Exclusion**			
Social defeat (range 20–80)	55	54	16
Perceived discrimination (range 9–36)	20	20	6,7
Social support (Z-scores range -4,32–4,88)	0.016	-0,096	2,3

Measures: K10 (depressive symptoms); PQ-16 (psychotic experiences); Social Defeat Scale; Everyday Discrimination Scale, Oslo Social Support Questionnaire.

‘lower education’ (no diploma, primary school, (preparation) lower and intermediate vocational education); ‘higher education’ (secondary education, higher vocational education, university).

**Fig 2:**
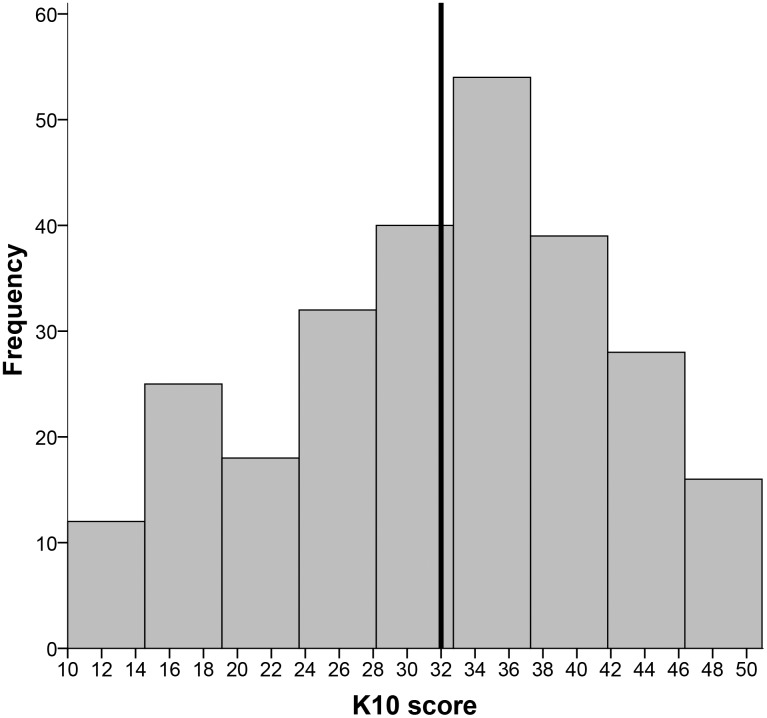
Distribution of depressive symptoms.

**Fig 3:**
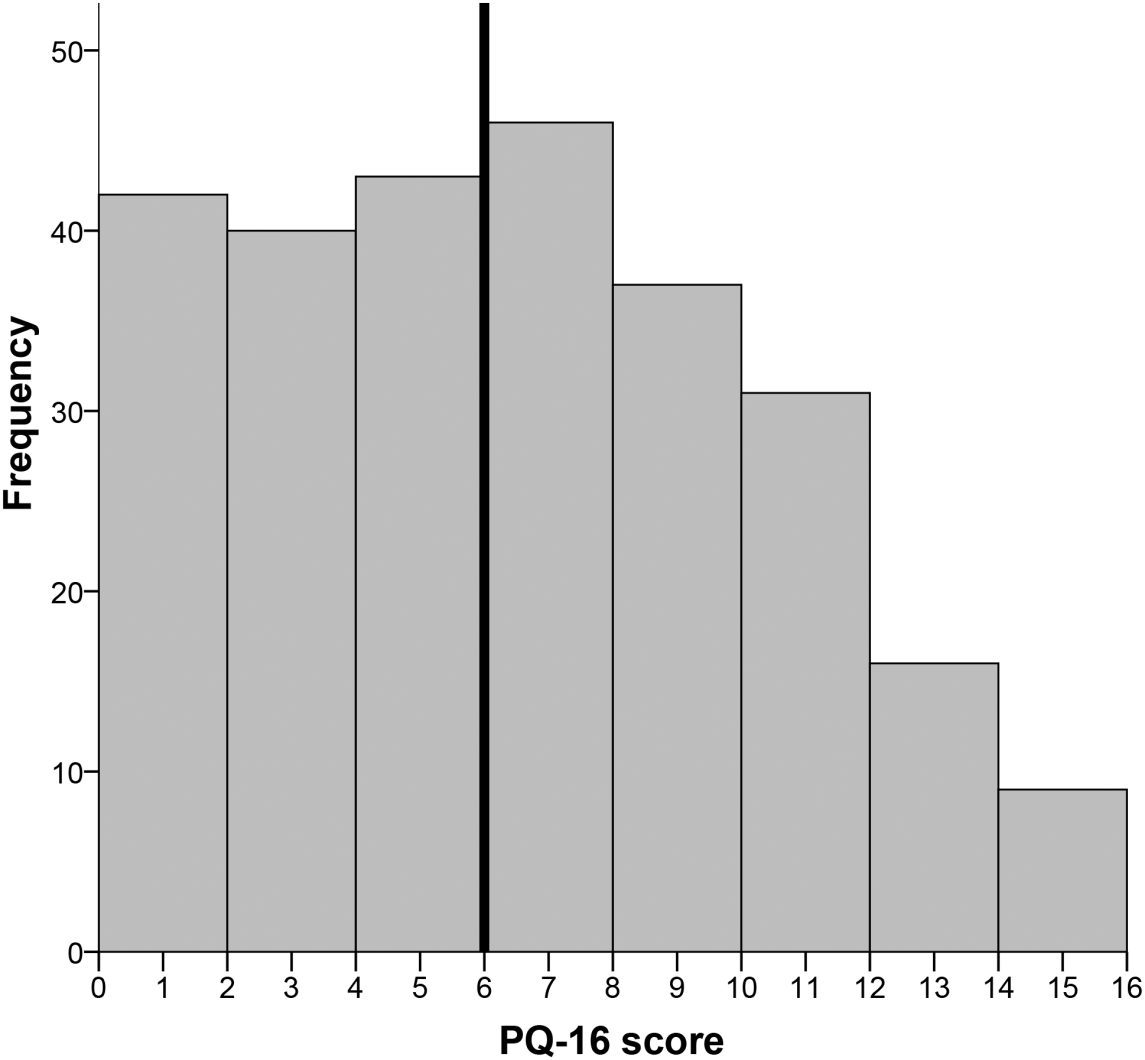
Distribution of psychotic experiences.

The median score on the K10 (depressive symptoms) was 33, which means that over 50% of the sample scores above the cut-off for depression, see Fig 2. The median score for the PQ-16 (psychotic experiences) was 6, which means that 50% scores above the cut-off for psychotic experiences, see Fig 3. Pearson correlation analysis showed a high correlation between depressive symptoms and psychotic experiences (r = .551, p <0.01).

### Social exclusion and psychiatric symptoms

Reports of social defeat and perceived discrimination were associated with more psychotic and depressive symptoms. Higher level of social support, on the other hand, predicted less psychotic experiences and depressive symptoms, see Table 2.

**Table 2:** Results of regression analyses into the associations between psychiatric symptoms and experiences of social exclusion.

Psychotic experiences				Depressive symptoms			
**social support**	**predictor**	**beta**	**adjusted R**^**2**^	**social support**	**predictor**	**beta**	**adjusted R**^**2**^
model 1***	social support	-0.252***	0.060	model 1***	social support	-0.383***	0.143
model 2***	social support	-0.221***	0.148	model 2***	social support	-0.363***	0.180
	education	-0.310***			education	-0.184**	
					age	0.156*	
**social defeat**				**social defeat**			
model 1***	social defeat	0.535***	0.284	model 1***	social defeat	0.725***	0.524
model 2***	social defeat	0.490***	0.334	model 2***	social defeat	0.718***	0.545
model 3***	social defeat	0.503***	0.332	model 3***	social defeat	0.708***	0.543
	social support	0.024			social support	-0.020	
	education	-0.226***			age	0.183***	
					first/second generation	0.113*	
**perceived discrimination**				**perceived discrimination**			
model 1***	discrimination	0.257***	0.062	model 1**	discrimination	0.179***	0.028
model 2***	discrimination	0.251***	0.161	model 2**	discrimination	0.187**	0.082
model 3***	discrimination	0.197**	0.179	model 3***	discrimination	0.070	0.181
	social support	-0.154*			social support	-0.339***	
	education	-0.316***			education	-0.186**	
					age	0.159*	
				Interaction analysis	Discrimination x social support	0.077#	0.176

model 1: unadjusted;

model 2: adjusted for age, gender, education, first/second generation migrant;

model 3: adjusted + social support

* p < 0.05;

** p < 0.01;

*** p < 0.001;

^#^ p = 0.670

When demographic variables were added to the regression models, associations between the social exclusion factors (social defeat, discrimination and social support) and symptoms (psychotic experiences and depressive symptoms) all remained statistically significant. Higher education reduced the impact of social exclusion on levels of symptoms, higher age contributed to associations between all social exclusion factors and depressive symptoms. Compared to first generation migrants, being a second-generation migrant was a significant factor in the regression model of social defeat as predictor of depressive symptoms.

Adding social support to the model did not change the association between social defeat and depressive symptoms nor the association between social defeat and psychotic experiences. Social support was statistically significant in the regression model of perceived discrimination and psychotic experiences. The explained variance improved slightly (adjusted R^2^ = 0.179 with social support versus 0.161 without, R^2^ change 0.021, p = 0.013) and the beta coefficient for discrimination decreased somewhat (0.197 with social support versus 0.251). The association between perceived discrimination and depressive symptoms was not significant anymore when social support was added, and the explained variance of the model improved (adjusted R^2^ = 0.181 with social support versus 0.082 without, R^2^ change 1.000, p < 0.001). We added an interaction term for discrimination and social support to the model. There was no moderation effect of social support on the relationship between discrimination and depressive symptoms.

### Social comparison and acculturation

The additional questionnaires about social comparison and acculturation were completed by a subset of 111 participants. Compared to the participants who did not continue with the additional questions, they were older (26 versus 23 years, p <0.01). There were no significant differences in gender, previous treatment, depressive symptoms and psychotic experiences.

Integration was the most common acculturation style (N = 38, 34%), followed by segregation (N = 32, 29%) assimilation (N = 27, 24%), and marginalization (N = 14, 13%). Levels of depressive symptoms and psychotic experiences did not differ significantly between acculturation styles.

On the social comparison scale, a lower score means a more negative self-image compared to Dutch natives. In the regression analysis with the K10 score, a negative self-image was associated with more depressive symptoms, also when adjusted for demographic parameters (beta -0.306; p = 0.001). Both social support (beta -0.207; p = 0.021) and education (beta -0.272; p = 0.001) were associated with less depressive symptoms. In the regression analysis with the PQ-16 score, a more negative self-image was associated with psychotic experiences (beta -0.204, p = 0.019), but this effect disappeared when social support was added to the model, although social support itself was also not significantly associated. Only education remained a significant protective factor in the analysis for psychotic experiences (beta -0.354; p < 0.001). Results of the regression analysis are shown in Table 3.

**Table 3:** Results of regression analysis into the associations between psychotic experiences, depressive symptoms and social comparison.

	N	Median	Mean	Std. dev.
**Social comparison**	118	63	63,1	22,2
*Regression analyses*				
**Psychotic experiences**	**predictor**		**beta**	**adjusted R**^**2**^
model 1***			-0.267**	0.063
model 2***			-0.204*	0.179
model 3***	social comparison		-0.138	0.193
	higher education		-0.359***	
			
**Depressive symptoms**	**predictor**		**beta**	**adjusted R**^**2**^
model 1***			-0.422***	0.171
model 2***			-0.391***	0.267
model 3***	social comparison		-0.306**	0.295
	higher education		-0.272**	
	social support		-0.207*	

model 1: unadjusted;

model 2: adjusted for age, gender, education, first/second generation migrant;

model 3: adjusted + social support

* p < 0.05;

** p < 0.01;

*** p < 0.001

### Sensitivity analysis

We compared the measures of social exclusion between the groups with and without previous mental healthcare. There were no significant differences in the scores on social defeat, discrimination and social support, suggesting that levels of social exclusion were not a consequence of any mental health problems. The regression analyses were performed again, using split file, based on previous use of mental healthcare. For all models, the adjusted R^2^-value was higher in the group without a history of previous mental healthcare, compared to the group with previous mental healthcare. This shows that the models have a better fit in the treatment-naive group. Furthermore, the betas of the explaining variables did not change in the group without a history of previous mental healthcare.

## Discussion

In this online study, over 50% of young Moroccan-Dutch young adults reported depressive symptoms and 50% reported psychotic experiences. Social defeat and perceived discrimination were associated with more psychotic experiences, and social defeat was associated with more depressive symptoms. Social support and higher level of education were associated with lower level of depressive symptoms and psychotic experiences. However, social support did not decrease the effect of perceived discrimination and social defeat.

The rates of reported psychiatric symptoms are high. This is especially remarkable for psychotic experiences, since 87% of the studied participants were women, whereas the increased incidence of psychotic disorders in migrants has to our knowledge only been reported in men [3]. The high rates of psychiatric symptoms suggest that we reached people who may have a need for (professional) support. Since the Moroccan-Dutch population is already at increased risk for developing psychiatric disorders, early intervention for psychiatric symptoms might have important beneficial effects. However, while the measures that we used, the K10 and PQ-16, are valid screening instruments for depressive disorder and for risk of psychotic disorder respectively, they do not give enough information to assess actual need for care. To overcome this, a high score on the K10 and PQ-16 instruments, combined with another instrument that can assess the needs for care, might enable online identification of individuals that would benefit from intervention. For psychotic experiences, we checked whether it would be more informative to (also) use the impact scale of the PQ-16, but this did not change results.

Social support was a protective factor for both depressive symptoms and psychotic experiences, but it did not diminish the effect of the risk factors. This is in line with two overview articles, which reported that social support in most studies did not have a buffering effect on the association between perceived discrimination and depressive symptoms [47,48], although it did have a protective effect on depression itself [48]. A recent study found a buffering effect for having many same-ethnicity friends in several populations, but not in Moroccan-Dutch migrants [21]. The online forum website Marokko.nl itself can also be a source of social support and is sometimes used to share private stories and receive support. Although this will help some people with mild symptoms, it will probably not be a sufficient intervention for people with more severe symptoms or who experience more negative social factors, like discrimination.

Higher education was a protective factor for both depressive symptoms and psychotic experiences. In previous studies, lower level of education was one of the indicators of social disadvantage which was associated with psychotic disorder [49] and was associated with higher level of depressive and/or anxiety disorders [50]. This finding is not limited to mixed or migrant populations [51,52]. Our interpretation is that a higher level of education is associated with better social integration (starting in school), better job perspectives, and therefore less social exclusion.

We found high rates of psychiatric symptoms in this sample (with 87% being females). Although Moroccan-Dutch people in general are not underrepresented in mental healthcare, there are gender differences. Moroccan-Dutch males use mental healthcare more often than Dutch males, whereas Moroccan-Dutch females use mental healthcare less often then Dutch females [53]. In a study into the perceived mental healthcare needs in migrants, Moroccan-Dutch people reported a low perceived need for regular care. The suggested explanation is that the Moroccan-Dutch population is a relatively conservative community, in which mental healthcare problems are more often attributed to traditional explanations, such as being possessed by a spirit, and help is also sought from traditional healers [54]. In a qualitative study in Israel, it was found that young Moroccan females felt they were part of the Western society, but that they suffered from an internal conflict between this western world and their traditional background. This increased their fear for stigma, and consequently decreased the probability that they would seek psychiatric help [55].

Given the high burden of psychiatric symptoms and the mismatch between mental healthcare and Moroccan-Dutch females, this population can benefit from an alternative way of offering help. Since we recruited our sample in an online community, it is logical to use the Internet to reach this at risk population for treatment purposes. Furthermore, the online environment is anonymous, which diminishes the fear for stigmatisation. On the website Marokko.nl, people indeed anonymously discuss mental health problems. We therefore created an add-on website to Marokko.nl, to deliver information about psychiatric disorders and traditional explanations, like being possessed, self-tests for depression and substance abuse and provided email contacts with healthcare workers and specially trained Imams [56]. The online setting enabled us to create this platform with a culturally sensitive look-and-feel, which helps to connect with the target population. Also for future interventions, this may help to fit the needs of Moroccan-Dutch females better. An online intervention would best fit with our results when it aims to increase the resilience for negative social factors, increase the access to social resources, and improve the social identity of participants. Since anonymity is important, we recommend an ‘online-only’ e-health intervention, in contrast to the frequent finding that blended care has better results [57–59]. An example is the web-based intervention ‘GET.ON Mood Enhancer Prevention training’, which consists of six interactive sessions and an online trainer [60]. Another possibility is mindfulness training, which focusses on increasing resilience in many different situations and problems and can be performed without guidance [61]. A one-session only intervention could be based on online contact with mental healthcare workers (chat, WhatsApp or email), in which the mental healthcare worker can explore the problems and social context of the person and deliver tailored support.

Our study has several strengths and limitations. A strength of our study is that we used different measures of social exclusion to investigate their association with depressive symptoms and psychotic experiences. This resulted in associations with great consistency: all in the same, hypothesized, direction. Our results in this online population extend on previous studies, which have described the link between social factors and psychotic disorder or depression in conventional study designs. Our findings should be viewed in the context of several limitations. Most of them are related to the online recruitment strategy. We collected a convenience sample and the cohort was collected based on self-selection, which leads to bias. Recruitment with the depression self-test probably will attract participants with depressive symptoms. Indeed, we found that the K10 score was significantly higher in the self-test subgroup compared to the research subgroup (35 vs 28). However, this means that also in the other group, 37% scored above the cut-off for depressive symptoms. Differences between the recruitment strategies in all variables are presented in the supplemental material, see S1 Table. Another example of self-selection is that 26% of the participants has received psychiatric treatment. In a Danish study, the cumulative risk for psychiatric treatment was 23% for males and 25% for females at age 50 [62]. In our study the mean age is 24,5 years, meaning that people with a history of psychiatric treatment are overrepresented in our sample. Another example of self-selection bias in our sample is that 87% of our participants were female (compared to 51% of the Marokko.nl users). Underrepresentation of males is reported in several other [63–66], but not all [26] online (mental health) studies. Women may be more interested in this type of research, but it cannot be excluded that Moroccan-Dutch males are for some reason less likely to participate in online mental health surveys than Dutch males would be. Interestingly, previous studies have reported high levels of psychopathology in Moroccan-Dutch men and not in Moroccan-Dutch women for psychotic disorder [3,67] and depressive disorder. High rates of psychiatric symptoms within the female Moroccan-Dutch population are thus a new finding. Furthermore, the 267 participants are just a small percentage of the > 2000 people who filled out the depression self-test and these 267 samples reflect 40% of the people who gave informed consent for the study. This self-selection bias decreases the generalizability of our study results. With online recruitment, it is not possible to collect a probability sample [68]. Incidence or prevalence figures can therefore not be calculated; we can only report that psychopathology scores are high, compared to cut-off scores from the literature. Furthermore, the PQ-16 for psychotic experiences was not designed for a migrant population, and not for a population that is not help-seeking. This can affect the interpretation of items by participants and the cut-off score, possibly leading to an overestimation of the psychotic experiences in our cohort. In our cross-sectional study, we did not collect information about the temporal relationship between social exclusion and psychopathology. We are therefore not able to prove causality. However, some longitudinal studies have shown that perceived discrimination precedes depression [69–71]. Furthermore, we compared the group with and without previous psychiatric treatment in the sensitivity analysis and we did not find evidence for reversed causality.

Due to the quantitative nature of our study, we have limited information about the mechanism behind the identified associations. We would like to know how participants reflect on the negative effect of social exclusion on their mental wellbeing, since awareness of this association could be the first step to increase empowerment and reduce the disease burden. We therefore are currently preparing a qualitative study, to further explore the perception of participants about the association between social exclusion and psychopathology.

Our study shows that the Moroccan-Dutch experience social exclusion and that this experience is related to their mental wellbeing. Within the Dutch society, there are currently strong antimigrant and anti-Muslim sentiments, which increase feelings of social exclusion. Furthermore, when psychopathology is already present, stigmatisation can result in further social exclusion. Preventive measures should aim to improve the development of a positive social identity in ethnic minorities, given the current negative social context. On the society level, we would like to emphasize the importance of social cohesion to improve mental wellbeing. Ethnic minority organisations can enhance social cohesion within migrant populations as well as between ethnic groups in the Netherlands, which can be stimulated by governmental funding programmes. Finally, it is important to stimulate initiatives to destigmatise mental health problems. An important measure to decrease stigma is delivering adequate information and approachable support, adapted to the migrant population.

## Supporting information

S1 Table Differences in variable scores between recruitment strategies. (DOCX)

S1 File Dutch survey questions. (PDF)

S2 File English survey questions. (DOCX)
