# Supplementary material for: Social exclusion and psychopathology in an online cohort of Moroccan-Dutch migrants: Results of the MEDINA-study
Source: PLoS One. 2017 Jul 10;12(7):e0179827. doi: 10.1371/journal.pone.0179827 (PMC5503196; doi:10.1371/journal.pone.0179827)
Supplement: S1 Table — (DOCX) [file pone.0179827.s001.docx]

**S1 Table, Differences in variable scores between recruitment strategies.**

|  | Recruitment route | Mean | N | Std. Deviation | ANOVA  p-value |
| --- | --- | --- | --- | --- | --- |
| Discrimination | Research | 19.6703 | 91 | 6.88324 | 0.405 |
|  | Self-test | 19.8303 | 165 | 6.30875 |  |
|  | Mailing | 21.7273 | 11 | 10.20873 |  |
| K10 score | Research | 27.5604 | 91 | 10.08101 | <0.001 |
|  | Self-test | 34.6380 | 163 | 8.44066 |  |
|  | Mailing | 31.7000 | 10 | 7.04036 |  |
| PQ16 score | Research | 5.2308 | 91 | 4.32583 | 0.057 |
|  | Self-test | 6.3636 | 165 | 3.67770 |  |
|  | Mailing | 8.3636 | 11 | 5.12392 |  |
| age | Research | 24.1685 | 89 | 6.82615 | <0.001 |
|  | Self-test | 24.0552 | 163 | 5.82380 |  |
|  | Mailing | 35.2222 | 9 | 10.99747 |  |
| Social Defeat | Research | 46.3736 | 91 | 15.73224 | <0.001 |
|  | Self-test | 58.1333 | 165 | 13.65852 |  |
|  | Mailing | 51.3636 | 11 | 18.95929 |  |
| Social support | Research | .4056 | 91 | 2.19817 | 0.005 |
|  | Self-test | -.4583 | 165 | 2.16016 |  |
|  | Mailing | 1.1888 | 11 | 3.41707 |  |
