# Supplementary material for: Social exclusion and psychopathology in an online cohort of Moroccan-Dutch migrants: Results of the MEDINA-study
Source: PLoS One. 2017 Jul 10;12(7):e0179827. doi: 10.1371/journal.pone.0179827 (PMC5503196; doi:10.1371/journal.pone.0179827)
Supplement: S1 File — (PDF) [file pone.0179827.s002.pdf]

Informed consent

Onderzoek naar sociale factoren en psychische klachten bij Marokkaanse Nederlanders.

Waarom dit onderzoek?
Uit onderzoek is gebleken dat psychische problemen vaker voorkomen bij migranten en hun kinderen. Ook is gebleken dat factoren in de samenleving daar invloed op hebben. Om hier meer inzicht in te krijgen willen we zoveel mogelijk Marokkaans-Nederlandse mensen vanaf 18 jaar vragen een vragenlijst in te vullen.

Hoe werkt het?
De vragenlijst gaat over psychische klachten en sociale factoren. De lijst kan online worden ingevuld en duurt ongeveer 10 minuten. Aan het eind van het onderzoek krijg je een aantal resultaten van jouw vragenlijst te zien. Als hieruit blijkt dat het verstandig kan zijn hulp te zoeken is daarover informatie beschikbaar. De ingevulde vragenlijsten worden geheel anoniem voor onderzoek verwerkt. De gegevens zullen op geen enkele manier naar een bepaalde persoon herleidbaar zijn.

Als je vragen over het onderzoek wilt stellen aan de onderzoeker kan dat via dit e-mail adres:
m.h.van.de.beek@umcg.nl.(Madelien van de Beek).

Voor dit onderzoek is de fase van deelnemers verzamelen afgerond. U kunt niet meer deel uitmaken van de studie. U kunt de vragenlijst wel nog voor uw eigen interesse invullen.

Meer weten? klik hier om meer informatie te downloaden.

Toestemming

- ◯ ik ben akkoord met deelname aan de studie
- ◯ nee, ik doe niet mee

Mijn leeftijd is boven de 18 jaar

- ◯ ja
- ◯ nee

Heb je deze vragenlijst al een keer eerder beantwoord?

- ◯ ja
- ◯ nee

K10

0% voltooid

De volgende vragen gaan over hoe je je voelde in de afgelopen 30 dagen. Vink per vraag het hokje aan dat het beste omschrijft hoe vaak je dit gevoel hebt.

Gedurende die afgelopen maand, hoe vaak voelde je je...

	Hele tijd	Meestal	Soms	Af en toe	Nooit
... erg vermoeid zonder duidelijke reden?	◯	◯	◯	◯	◯
... zenuwachtig?	◯	◯	◯	◯	◯
... zo zenuwachtig dat niets je tot rust kon brengen?	◯	◯	◯	◯	◯
... hopeloos?	◯	◯	◯	◯	◯
... rusteloos of ongedurig?	◯	◯	◯	◯	◯
... zo rusteloos dat je niet meer stil kon zitten?	◯	◯	◯	◯	◯
... somber of depressief?	◯	◯	◯	◯	◯
... zo somber dat niets je kon opvrolijken?	◯	◯	◯	◯	◯
... zo, dat alles veel moeite kostte?	◯	◯	◯	◯	◯
... waardeloos?	◯	◯	◯	◯	◯

PQ-16

8% voltooid

Hieronder staan een aantal uitspraken over je gedachten, gevoelens en ervaringen. Lees alsjeblieft eerst elke uitspraak zorgvuldig en vink dan aan of je het ermee 'eens' of 'oneens' bent.

	eens	oneens
1. Ik voel me ongeïnteresseerd in de dingen die ik vroeger leuk vond.	◯	◯
2. Ik ben vaak in een situatie, waarvan ik het gevoel heb dat het precies zo al eerder is gebeurd (déjà vu).	◯	◯
3. Ik ruik of proef soms dingen die anderen niet kunnen ruiken of proeven.	◯	◯
4. Ik hoor vaak ongewone geluiden zoals gebonk, getik, gesis, geklap, of gerinkel in mijn oren.	◯	◯
5. Ik haal wel eens door elkaar of iets dat ik heb meegemaakt echt was of fantasie.	◯	◯
6. Wanneer ik iemand aankijk, of mijzelf in de spiegel bekijk, heb ik het gezicht vlak voor mijn ogen zien veranderen.	◯	◯
7. Ik raak buitengewoon ongerust als ik mensen voor het eerst ontmoet.	◯	◯
8. Ik heb dingen gezien die andere mensen blijkbaar niet kunnen zien.	◯	◯
9. Mijn gedachten zijn soms zo sterk dat ik ze bijna kan horen.	◯	◯
10. Ik zie soms speciale betekenissen in advertenties, etalages of in de manier waarop dingen om mij heen zijn opgesteld.	◯	◯
11. Soms heb ik het gevoel gehad dat ik niet de baas was over mijn eigen ideeën of gedachten.	◯	◯

12. Soms voel ik me plotseling afgeleid door geluiden van veraf, die ik gewoonlijk niet bemerk.		
13. Ik heb dingen gehoord die andere mensen niet horen, zoals stemmen van fluisterende of pratende mensen.		
14. Ik heb vaak het gevoel dat anderen mij kwaad willen doen.		
15. Ik heb het gevoel gehad dat er een of andere persoon of kracht om mij heen is, ook al kan ik niemand zien.		
16. Ik heb het gevoel dat sommige delen van mijn lichaam op een of andere manier veranderd zijn of dat delen van mijn lichaam anders werken dan voorheen.		

16% voltooid

Onderstaande uitspraken heb je met "eens" beantwoord. Wil je per uitspraak aangeven hoeveel last je ervan ondervindt?

	geen last	een beetje last	nogal wat last	veel last
» 1. Ik voel me ongeïnteresseerd in de dingen die ik vroeger leuk vond.				
» 2. Ik ben vaak in een situatie, waarvan ik het gevoel heb dat het precies zo al eerder is gebeurd (déjà vu).				
» 3. Ik ruik of proef soms dingen die anderen niet kunnen ruiken of proeven.				
» 4. Ik hoor vaak ongewone geluiden zoals gebonk, getik, gesis, geklap, of gerinkel in mijn oren.				
» 5. Ik haal wel eens door elkaar of iets dat ik heb meegemaakt echt was of fantasie.				
» 6. Wanneer ik iemand aankijk, of mijzelf in de spiegel bekijk, heb ik het gezicht vlak voor mijn ogen zien veranderen.				
» 7. Ik raak buitengewoon ongerust als ik mensen voor het eerst ontmoet.				
» 8. Ik heb dingen gezien die andere mensen blijkbaar niet kunnen zien.				
» 9. Mijn gedachten zijn soms zo sterk dat ik ze bijna kan horen.				
» 10. Ik zie soms speciale betekenissen in advertenties, etalages of in de manier waarop dingen om mij heen zijn opgesteld.				
» 11. Soms heb ik het gevoel gehad dat ik niet de baas was over mijn eigen ideeën of gedachten.				
» 12. Soms voel ik me plotseling afgeleid door geluiden van veraf, die ik gewoonlijk niet bemerk.				
» 13. Ik heb dingen gehoord die andere mensen niet horen, zoals stemmen van fluisterende of pratende mensen.				
» 14. Ik heb vaak het gevoel dat anderen mij kwaad willen doen.				
» 15. Ik heb het gevoel gehad dat er een of andere persoon of kracht om mij heen is, ook al kan ik niemand zien.				
» 16. Ik heb het gevoel dat sommige delen van mijn lichaam op een of andere manier veranderd zijn of dat delen van mijn lichaam anders werken dan voorheen.				

Discriminatie schaal

24% voltooid

Hoe vaak in je leven werd je gediscrimineerd vanwege je huidskleur, land van herkomst, sekse, leeftijd, godsdienst, uiterlijk, seksuele oriëntatie of andere kenmerken - zoals de volgende voorbeelden:

	Nooit	1 keer	wel 2 of 3 keer	meer dan 4 keer	ik weet het niet
Een leraar raadde u af om hoger onderwijs te proberen					
Je kreeg geen baan					
Je kreeg geen promotie					
Je werd ontslagen					
Je kon geen huis huren of kopen in de buurt waar je dat graag wilde					
Je kon niet blijven wonen omdat de buren je het leven onaangenaam maakten					
Je werd lastig gevallen door de politie					
De bank gaf je geen lening of hypotheek					
Je kreeg slechte medische zorg					
Je kreeg slechte service van een loodgieter, automonteur of andere beroepskracht					

32% voltooid

Hoe vaak ervaar je nu één van de volgende typen van discriminatie?

	Nooit	Zelden	Soms	Vaak
Je wordt minder beleefd behandeld dan andere mensen				
Je wordt met minder respect behandeld dan andere mensen				

Je wordt minder goed bediend in een winkel of restaurant	○	○	○	○
Men doet alsof je dom bent	○	○	○	○
Men doet alsof je gevaarlijk bent	○	○	○	○
Men doet alsof je oneerlijk bent	○	○	○	○
Men doet alsof je minder bent	○	○	○	○
Je wordt uitgescholden of beledigd	○	○	○	○
Je wordt bedreigd of lastig gevallen	○	○	○	○

40% voltooid

Wat waren de belangrijkste redenen van de discriminatie die je meemaakte? (meerdere antwoorden mogelijk)

- ☐ Je leeftijd
- ☐ Je sekse
- ☐ Je huidskleur
- ☐ Je land van herkomst of nationaliteit
- ☐ Je religie
- ☐ Je lengte of gewicht
- ☐ Een ander aspect van je uiterlijk
- ☐ Een lichamelijk gebrek
- ☐ Je seksuele oriëntatie
- ☐ Een andere reden, namelijk: ______
- ☐ Nooit eerder discriminatie meegemaakt

44% voltooid

In welke mate heeft discriminatie je belemmerd in het leiden van een volwaardig en productief leven?

- ○ In hoge mate
- ○ In enige mate
- ○ Een klein beetje
- ○ Helemaal niet
- ○ Niet van toepassing

In welke mate is je leven moeilijker geweest ten gevolge van discriminatie?

- ○ In hoge mate
- ○ In enige mate
- ○ Een klein beetje
- ○ Helemaal niet
- ○ Niet van toepassing

Social defeat

48% voltooid

Hieronder vind je een aantal uitspraken die beschrijven hoe mensen tegen zichzelf aankijken.
Lees elke uitspraak aandachtig en vink het bolletje aan dat het best beschrijft hoe je je de afgelopen 7 dagen voelde.

	Nooit	Zelden	Soms	Meestal	Altijd
1. Ik heb het gevoel dat ik het gemaakt heb in het leven.	○	○	○	○	○
2. Ik voel me een succesvol persoon.	○	○	○	○	○
3. Ik voel me verslagen door het leven.	○	○	○	○	○
4. Ik voel me in oorsprong een winnaar.	○	○	○	○	○
5. Ik heb het gevoel dat ik mijn positie in de wereld ben verloren.	○	○	○	○	○
6. Ik heb het gevoel dat het leven me heeft behandeld als een voetveeg.	○	○	○	○	○
7. Ik voel me machteloos.	○	○	○	○	○
8. Ik voel dat het vertrouwen uit me is weggeslagen	○	○	○	○	○
9. Ik voel me in staat het hoofd te bieden aan wat me overkomt.	○	○	○	○	○
10. Ik heb het gevoel dat ik ben gedaald tot de onderste trede van de maatschappelijke ladder.	○	○	○	○	○
11. Ik voel me compleet lamgeslagen.	○	○	○	○	○
12. Ik voel me een verliezer.	○	○	○	○	○
13. Ik voel dat ik het opgegeven heb.	○	○	○	○	○
14. Ik voel me een mislukking.	○	○	○	○	○
15. Ik heb het gevoel dat ik belangrijke slagen in het leven heb verloren.	○	○	○	○	○
16. Ik voel dat er geen strijdlust meer in me zit.	○	○	○	○	○

Social support

56% voltooid

Als je nu ernstige persoonlijke problemen zou hebben, op hoeveel familie, vrienden, kennissen en dergelijke zou je dan echt kunnen

rekenen?

- geen
- 1 of 2
- 3 tot 5
- meer dan 5

Hoeveel betrokkenheid laten mensen je zien bij wat je doet?

- veel betrokkenheid
- enige betrokkenheid
- onzeker
- weinig betrokkenheid
- geen betrokkenheid

Hoe gemakkelijk is het voor je om praktische hulp van buren te krijgen als je die nodig zou hebben?

- heel gemakkelijk
- gemakkelijk
- mogelijk
- moeilijk
- erg moeilijk

Demografische gegevens

64% voltooid

Wat is je leeftijd?

Wat is je geslacht?

- man
- vrouw

In welk land ben je geboren?

- Nederland
- Marokko
- elders, namelijk:

In welk land is je moeder geboren?

- Nederland
- Marokko
- elders, namelijk:

In welk land is je vader geboren?

- Nederland
- Marokko
- elders, namelijk:

Hoe oud was je toen je naar Nederland verhuisde?

72% voltooid

Hoe belangrijk is religie voor jou?

niet belangrijk	enigszins belangrijk	redelijk belangrijk	heel belangrijk
○	○	○	○

Hoe vaak gebruik je drugs, anders dan alcohol?

Nooit	Een keer per maand of minder vaak	2-4 keer per maand	2-3 keer per week	4 keer per week of vaker
○	○	○	○	○

Gebruik je meer dan één type drugs bij dezelfde gelegenheid?

Nooit	Een keer per maand of minder vaak	2-4 keer per maand	2-3 keer per week	4 keer per week of vaker
○	○	○	○	○

Hoeveel keer neem je drugs op een dag dat je drugs gebruikt?

1-2	3-4	5-6	7 of meer
○	○	○	○

Welke drugs neem je meestal als je drugs gebruikt? (meerdere antwoorden zijn mogelijk)

- ☐ Cannabis (Hasj, Wiet, Space cake ed.)
- ☐ Amfetamines (Speed, Ritalin Christal, Pep, Methamfetamine ed.)
- ☐ Cocaïne
- ☐ Opïaten (Heroïne, Methadon, Opium ed.)
- ☐ Hallucinogenen (XTC, LSD, Ketamine, MDMA, PCP, Paddo's ed.)
- ☐ Oplosmiddelen (Benzine, Lijm, Thinner ed.)
- ☐ Overigen (anabole steroïden, Efedra, GHB, Ether, Herbals ed.)

Ben je eerder in behandeling geweest voor psychische klachten?

- ○ ja
- ○ nee

Wat was de diagnose of wat waren je klachten?

Gebruik je momenteel medicatie in verband met psychische klachten?

- ○ ja
- ○ nee

Welke medicatie is dat?

80% voltooid

Wat zijn de eerste 4 cijfers van je postcode?
(dit geeft informatie over de wijk waarin je woont, wat voor ons onderzoek interessant is. De eerste 4 cijfers van je postcode zijn niet aan een specifiek adres gekoppeld. Je anonimiteit blijft dus gewaarborgd)

Wat is je gemiddelde bruto maandelijkse inkomen?

Wat is je hoogst behaalde diploma?

maak je keuze:

Contact informatie

88% voltooid

Je zou ons heel erg helpen als je op een later moment (bijvoorbeeld volgend jaar) nog eens mee wilt doen aan een vragenlijst als deze. Mogen we je hiervoor benaderen?

- ○ ja
- ○ nee

Via welk email adres kunnen we je benaderen?

Wil je kans maken op de waardebon?

- ○ ja
- ○ nee

96% voltooid

Kunnen we je daarvoor bereiken op het email adres dat je hiervoor hebt ingevuld: ${q://QID81/ChoiceTextEntryValue}?

- ○ ja
- ○ nee, ander email adres:

Geef dan hier het email adres op waarop we je kunnen bereiken als je hebt gewonnen:

Heb je nog overige opmerkingen over de vragenlijst?

Feedback tekst

100% voltooid

Beste deelnemer,

Heel hartelijk bedankt voor je deelname!
Na elke 50ste deelnemer wordt er een waardebon verloot. De winnaars krijgen hiervan automatisch bericht.
Voor de geïnteresseerden zullen de uitkomsten van het totale onderzoek na afronding op de website ziekofbezeten.nl worden gepubliceerd. Bij overige vragen kun je contact opnemen via: m.h.van.de.beek@umcg.nl

Mede namens de overige onderzoekers, hartelijk dank,
Madelien van de Beek
arts onderzoeker UMCG

Hieronder vind je je persoonlijke score op de vragenlijst:

Depressieve klachten en angstklachten
Er waren 10 vragen in de lijst die klachten van depressie en angst meten. De laagst mogelijke score is 10 (helemaal geen klachten), de hoogst mogelijke score is 50 (zeer ernstige klachten).

Jij hebt de volgende score: ${gr://SC6/Score}

Vergelijk je score met de volgende beschrijvingen:
10 - 19 geen of minimale klachten
20 - 24 milde depressieve en/of angstklachten.
22 - 29 matige depressieve en/of angstklachten.
30 - 50 ernstige depressieve en/of angstklachten.

Als je een score hebt van boven de 30 en je herkent je in die omschrijving, adviseren wij je hulp te zoeken. Je kunt contact opnemen met je huisarts, of met hulpverleners waarvan de adressen te vinden zijn op de website www.ziekofbezeten.nl onder de knop "hulp&info". Op deze website vind je ook meer informatie over depressie en angst.

Psychotische klachten
Er zijn in de lijst 16 vragen die 'bijzondere' of 'psychotische' ervaringen meten. De laagst mogelijke score is 0 (geen bijzondere ervaringen), de hoogst mogelijke score is 16 (veel bijzondere ervaringen).

Jij hebt de volgende score: ${gr://SC7/Score}

Een score boven de 6 geeft aan dat je meer dan gemiddeld bijzondere/psychotische ervaringen meemaakt. Je hoeft hier niet van te schrikken en dit hoeft niets te betekenen. Als je zelf veel last hebt van deze ervaringen kun je contact opnemen met je huisarts, of met hulpverleners waarvan de adressen te vinden zijn op de website www.ziekofbezeten.nl onder de knop "hulp&info". Op de website vind je ook meer informatie over psychose.

Discriminatie
Op het gedeelte van de vragenlijst dat discriminatie meet, kunnen scores variëren tussen 19 (nooit discriminatie meegemaakt) en 84 (zeer veel ervaring met discriminatie). Hoe hoger jouw score is, hoe vaker je discriminatie hebt meegemaakt. Jouw score geeft inzicht hoe vaak je discriminatie hebt meegemaakt. We kunnen dit nog niet vergelijken met uitkomsten van ander onderzoek. Daarom kunnen we aan de score geen advies koppelen.

Jij hebt de volgende score: ${gr://SC9/Score}

Wil je nog meer soortgelijke vragen beantwoorden?

Dit is het einde van het basisgedeelte van de vragenlijst. Als je je email adres hebt ingevuld, kom je in aanmerking voor de waardebon en kun je het onderzoek nu afsluiten. Als je het leuk vindt om nog een aantal vragen te beantwoorden (ongeveer 5 minuten), kan je dat hieronder aangeven. Natuurlijk zou je ons daarmee een grote dienst bewijzen!

- ja, ik ben bereid om nog een aantal vragen in te vullen
- nee, ik heb geen interesse

Social Comparison

In vergelijking met autochtone Nederlanders van mijn leeftijd voel ik mij in het algemeen:

Inferieur (de mindere)	Superieur (de meerdere)
Onbekwaam	Bekwaam
Onaardig	Aardig
Buitengesloten	Geaccepteerd
Anders	Hetzelfde
Ongetalenteerd	Getalenteerd
Zwakker	Sterker
Onzeker	Zeker
Ongewenst	Gewenst
Onaantrekkelijk	Aantrekkelijk
Outsider (buitenstaander)	Insider (iemand die erbij hoort)

Acculturation

Hoe belangrijk vind je het om volgens de Marokkaanse normen en waarden te leven?

Erg belangrijk	Belangrijk	Neutraal	Onbelangrijk	Erg onbelangrijk

Hoe belangrijk vind je het om Nederlandse vrienden en kennissen te hebben?

Erg belangrijk	Belangrijk	Neutraal	Onbelangrijk	Erg onbelangrijk

Hoe voel je je van binnen?

- Overwegend Marokkaans

Marokkaans-Nederlands

Overwegend Nederlands

Geen van beiden

Geef bij de volgende uitspraken je mening.

	Helemaal mee oneens	Redelijk mee oneens	Neutraal	Redelijk mee eens	Helemaal mee eens
Met vrienden spreek ik veel Nederlands					
Met vrienden spreek ik veel Marokkaans					
Thuis spreek ik veel Nederlands					
Thuis spreek ik veel Marokkaans					
Ik heb veel Nederlandse vrienden					
Ik heb veel Marokkaanse vrienden in Nederland					
Ik heb veel Marokkaanse vrienden in Marokko					
Ik vier Nederlandse feestdagen					
Ik vier Marokkaanse feestdagen					
Ik vind Nederlandse kleding mooi					
Ik vind Marokkaanse kleding mooi					
Ik vind het belangrijk om het Nederlandse nieuws te volgen					
Ik vind het belangrijk om het Marokkaanse nieuws te volgen					

DUDIT

Hier volgen een aantal vragen over drugs. De vragen gaan over de periode van het afgelopen jaar.

	Nooit	Minder vaak dan een keer per maand	Elke maand	Elke week	Dagelijks of bijna dagelijks
Hoe vaak ben je zwaar onder invloed van drugs?					
Hoe vaak was je verlangen naar drugs zo sterk dat je dit niet kon weerstaan?					
Is het voorgekomen dat je niet meer kon stoppen met het gebruik van drugs nadat je was begonnen?					
Hoe vaak heb je drugs gebruikt en toen nagelaten iets te doen dat je zou doen?					
Hoe vaak heb je 's ochtends een drug moeten gebruiken na zwaar druggebruik de dag ervoor?					
Hoe vaak heb je schuldgevoelens of een slecht geweten gehad omdat je drugs gebruikte?					

Ben jij, of is iemand anders wel eens gewond geraakt (geestelijk of lichamelijk) omdat je drugs gebruikte?

Nee   Ja, maar niet in het afgelopen jaar   Ja, in het afgelopen jaar

Heeft een familielid of vriend, een dokter of een verpleegkundige, of iemand anders zich zorgen gemaakt om je druggebruik of tegen je gezegd dat je zou moeten stoppen met het gebruiken van drugs?

Nee   Ja, maar niet in het afgelopen jaar   Ja, in het afgelopen jaar
