# Supplementary material for: Social exclusion and psychopathology in an online cohort of Moroccan-Dutch migrants: Results of the MEDINA-study
Source: PLoS One. 2017 Jul 10;12(7):e0179827. doi: 10.1371/journal.pone.0179827 (PMC5503196; doi:10.1371/journal.pone.0179827)
Supplement: S2 File — (DOCX) [file pone.0179827.s003.docx]

**S2 File, Survey – English translation**

**Depressive symptoms –** *K10*

**
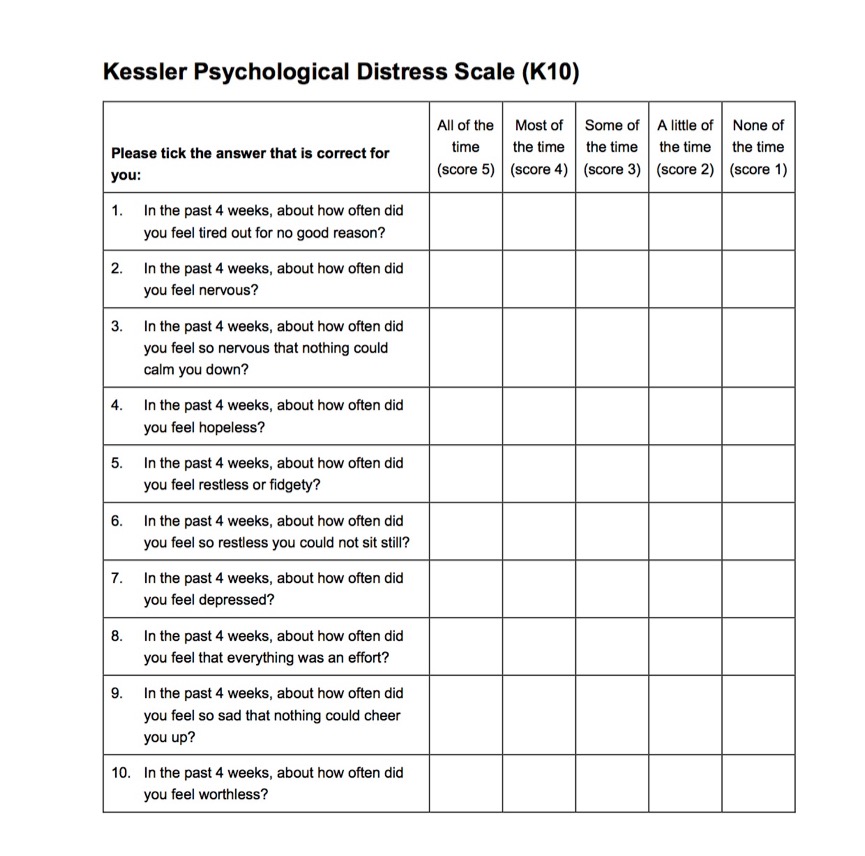
**

*reference:*

Kessler RC, Barker PR, Colpe LJ, Epstein JF, Gfroerer JC, Hiripi E, et al. Screening for serious mental illness in the general population. Arch Gen Psychiatry. 2003 Feb;60(2):184-9.

**Psychotic experiences–** *PQ-16*

| **About the Protocol** | |
| --- | --- |
| **Description of Protocol:** | The Prodromal Questionnaire–16 (PQ-16) includes 16 self-reported true/false items that screen for the risk of psychosis. Nine items assess perceptual abnormalities and hallucinations, five items assess unusual thought content, delusional ideas and paranoia, and two items assess negative symptoms. |
| **Protocol Text:** | 1. I feel uninterested in the things I used to enjoy.  [ ] True  [ ] False  1.1. If True: how much distress did you experience?  [ ] 0 No  [ ] 1 Mild  [ ] 2 Moderate  [ ] 3 Severe  2. I often seem to live through events exactly as they happened before (déjà vu.)  [ ] True  [ ] False  2.1. If True: how much distress did you experience?  [ ] 0 No  [ ] 1 Mild  [ ] 2 Moderate  [ ] 3 Severe  3. I sometimes smell or taste things that other people can’t smell or taste.  [ ] True  [ ] False  3.1. If True: how much distress did you experience?  [ ] 0 No  [ ] 1 Mild  [ ] 2 Moderate  [ ] 3 Severe  4. I often hear unusual sounds like banging, clicking, hissing, clapping or ringing in my ears.  [ ] True  [ ] False  4.1. If True: how much distress did you experience?  [ ] 0 No  [ ] 1 Mild  [ ] 2 Moderate  [ ] 3 Severe  5. I have been confused at times whether something I experienced was real or imaginary.  [ ] True  [ ] False  5.1. If True: how much distress did you experience?  [ ] 0 No  [ ] 1 Mild  [ ] 2 Moderate  [ ] 3 Severe  6. When I look at a person, or look at myself in a mirror, I have seen the face change right before my eyes.  [ ] True  [ ] False  6.1. If True: how much distress did you experience?  [ ] 0 No  [ ] 1 Mild  [ ] 2 Moderate  [ ] 3 Severe  7. I get extremely anxious when meeting people for the first time.  [ ] True  [ ] False  7.1. If True: how much distress did you experience?  [ ] 0 No  [ ] 1 Mild  [ ] 2 Moderate  [ ] 3 Severe  8. I have seen things that other people apparently can’t see.  [ ] True  [ ] False  8.1. If True: how much distress did you experience?  [ ] 0 No  [ ] 1 Mild  [ ] 2 Moderate  [ ] 3 Severe  9. My thoughts are sometimes so strong that I can almost hear them.  [ ] True  [ ] False  9.1. If True: how much distress did you experience?  [ ] 0 No  [ ] 1 Mild  [ ] 2 Moderate  [ ] 3 Severe  10. I sometimes see special meanings in advertisements, shop windows, or in the way things are arranged around me.  [ ] True  [ ] False  10.1. If True: how much distress did you experience?  [ ] 0 No  [ ] 1 Mild  [ ] 2 Moderate  [ ] 3 Severe  11. Sometimes I have felt that I’m not in control of my own ideas or thoughts.  [ ] True  [ ] False  11.1. If True: how much distress did you experience?  [ ] 0 No  [ ] 1 Mild  [ ] 2 Moderate  [ ] 3 Severe  12. Sometimes I feel suddenly distracted by distant sounds that I am not normally aware of.  [ ] True  [ ] False  12.1. If True: how much distress did you experience?  [ ] 0 No  [ ] 1 Mild  [ ] 2 Moderate  [ ] 3 Severe  13. I have heard things other people can’t hear like voices of people whispering or talking.  [ ] True  [ ] False  13.1. If True: how much distress did you experience?  [ ] 0 No  [ ] 1 Mild  [ ] 2 Moderate  [ ] 3 Severe  14. I often feel that others have it in for me.  [ ] True  [ ] False  14.1. If True: how much distress did you experience?  [ ] 0 No  [ ] 1 Mild  [ ] 2 Moderate  [ ] 3 Severe  15. I have had the sense that some person or force is around me, even though I could not see anyone.  [ ] True  [ ] False  15.1. If True: how much distress did you experience?  [ ] 0 No  [ ] 1 Mild  [ ] 2 Moderate  [ ] 3 Severe  16. I feel that parts of my body have changed in some way, or that parts of my body are working differently than before.  [ ] True  [ ] False  16.1. If True: how much distress did you experience?  [ ] 0 No  [ ] 1 Mild  [ ] 2 Moderate  [ ] 3 Severe  **Scoring:**  Respondents that endorse 6 symptom items or more are considered to be at risk for psychosis. |
| **Participant:** | Adolescents and adults, ages 12 and older |
| **Source:** | Ising, H. K., Veling, W., Loewy, R. L., Rietveld, M. W., Rietdijk, J., Dragt, S., Klaassen, R. M. C., Neiman, D. H., Wunderink, L., Linszen, D. H., & van der Gaag, M. (2012). The validity of the 16-item version of the Prodromal Questionnaire (PQ-16) to screen for ultra high risk of developing psychosis in the general help-seeking population. *Schizophrenia Bulletin, 38*(6), 1288–1296. |
| **Personnel and Training Required:** | None |
| **Equipment Needs:** | None |
| **General References:** | Loewy, R. L., Bearden, C. E., Johnson, J. K., Raine, A., & Cannon, T. D. (2005). The prodromal questionnaire (PQ): Preliminary validation of a self-report screening measure for prodromal and psychotic syndromes. *Schizophrenia Research, 79*(1), 117–125.  van de Beek, M. H., van der Krieke, L., & Schoevers, R. A. (2014). Migrants Examined for Determinants of psychopathology through INternet Assessment (MEDINA) study: A cross-sectional study among visitors of an Internet community*. BMJ Open, 4*(1), e003980. |

**Discrimination –** *everyday discrimination scale & Major Experiences of Discrimination: 9 item version from the MIDUS Study*

In your day-to-day life, how often do any of the following things happen to you? 1. You are treated with less courtesy than other people are.

2. You are treated with less respect than other people are.

3. You receive poorer service than other people at restaurants or stores.

4. People act as if they think you are not smart.

5. People act as if they are afraid of you.

6. People act as if they think you are dishonest.

7. People act as if they’re better than you are.

8. You are called names or insulted.

9. You are threatened or harassed.

*response***:**

| Never | Seldom | Sometimes | Often |
| --- | --- | --- | --- |

How many times in your life have you been discriminated against in each of the

following ways because of such things as your race, ethnicity, gender, age, religion, physical appearance, sexual orientation, or other characteristics?

1. You were discouraged by a teacher or advisor from seeking higher education? 3. You were not hired for a job?
4. You were not given a job promotion?
5. You were fired?

6. You were prevented from renting or buying a home in the neighborhood you wanted?

7. You were prevented from remaining in a neighborhood because neighbors made life so uncomfortable?

8. You were hassled by the police

9. You were denied a bank loan?

10. You were denied or provided inferior medical care?

11. You were denied or provided inferior service by a plumber, car mechanic, or other service provider?

*response***:**

| Never | Once | 2-3 times | More than 4 times | I do not know |
| --- | --- | --- | --- | --- |

GLOBAL FOLLOW UP QUESTION AFTER ALL ITEMS:

What was the main reason for the discrimination you experienced? (If more than one main reason, circle all that apply.)

1. Your age

2. Your gender

3. Your race

4. Your ethnicity or nationality

5. Your religion

6. Your height or weight

7. Some other aspect of your appearance

8. A physical disability

9. Your sexual orientation

10. Some other reason for discrimination (Please specify:) ________________________________________________________________ ________________________________________________________________

How much has discrimination interfered with you having a full and productive life?

1. A lot

2. Some

3. A little

4. Not at all

5. not applicable

How much harder has your life been because of discrimination?

1. A lot

2. Some

3. A little

4. Not at all

5. not applicable

*references:*

- Kessler, R.C., Mickelson, K., and Williams, D.R. “The Prevalence, Distribution, and Mental Health Correlates of Perceived Discrimination in the United States.” Journal of Health and Social Behavior. 1999 40(3):208-230.
- Krieger N., Smith K., Naishadham D., Hartman C., Barbeau E.M. “Experiences of discrimination: validity and reliability of a self-report measure for population healthresearch on racism and health.” Social Science & Medicine. 2005; 61(7):1576-1596.
- Taylor T.R., Kamarck T.W., Shiffman S. “Validation of the Detroit area study discrimination scale in a community sample of older African American adults: the Pittsburgh healthy heart project.” International Journal of Behavioral Medicine. 2004; 11:88–94.

**Social defeat –** *social defeat schaal*

**
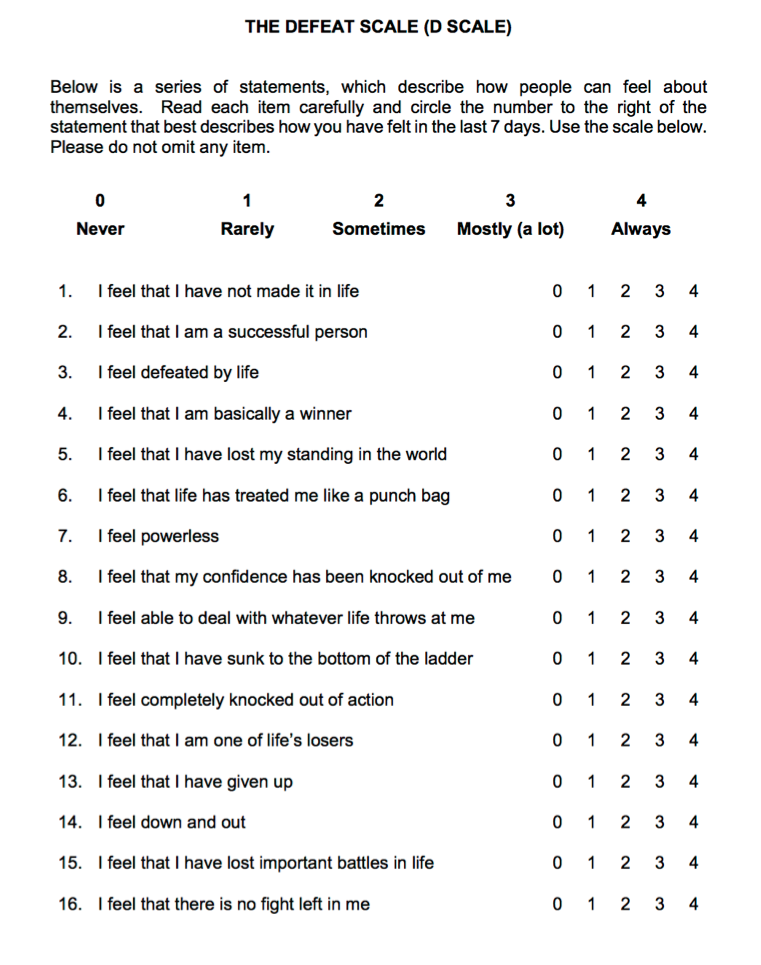
**

*Reference:*

- Gilbert, P. & Allan, S. (1998). The role of defeat and entrapment (arrested flight) in depression: An exploration of an evolutionary view. Psychological Medicine, 28, 585- 598.
- Gilbert, P., Allan, S., Brough, S., Melley, S., & Miles, J. (2002). Anhedonia and positive affect: relationship to social rank, defeat and entrapment. Journal of Affective Disorders, 71, 141-151.
- Gilbert, P. (2001a). Evolutionary approaches to psychopathology: The role of natural defences. Australian and New Zealand Journal of Psychiatry, 35, 17-27.
- Gilbert, P. (2001b). Depression and stress: A biopsychosocial exploration of evolved functions and mechanisms Stress: The International Journal of the Biology of Stress, 4, 121-135.
- Gilbert. P. (2007). Psychotherapy and Counselling for depression. London: Sage.

**Social Support –** *Oslo social support questionnaire*

# The Oslo 3-items social support scale

1. How easy can you get help from neighbours if you should need it?

(Very easy, easy, possible, difficult, very difficult)

1. How many people are so close to you that you can count on them if you have serious problems? (none, 1-2, 3-5, 5+)
2. How much concern do people show in what you are doing? (a lot, some, uncertain, little, no).

*References:*

- Dowrick,C, Casey,P, Dalgard, O et al. (1998). Outcomes of Depression International Network (ODIN). Br J Psychiat, 1998, 172, 359-363)
- Meltzer H (2003). Development of a common instrument for mental health. In: Nosikov & Gudex (eds). EUROHIS: Developing Common Instruments for Health Surveys. Amsterdam: IOS Press

**Demographic variables**

- What is your age?
- Wat is your gender? Male/female
- In which country are you born? Netherlands/Morocco/other
- In which country is your mother born? Netherlands/Morocco/other
- In which country is your father born? Netherlands/Morocco/other
- At what age did you move to the Netherlands (if applicable)
- How important is religion for you? Not important/somewhat important/rather important/very important
- Did you get previous treatment for psychic complaints? Yes/no
- What was the diagnosis? (if applicable)
- Do you currently use medication for psychic complaints? Yes/no
- What medication do you use? (if applicable)
- What are the first 4 digits of your postal code?
- What is your average monthly income?
- What is your highest qualification? (chose between options)
- Can we contact you again for further research?
- Via which email address? (if applicable)
- Do you want to participate in the voucher raffle?
- Via which email address? (if applicable)
- Do you have any remarks about the survey?

***Additional questionnaires***

**Social comparison** *– social comparison scale*

**
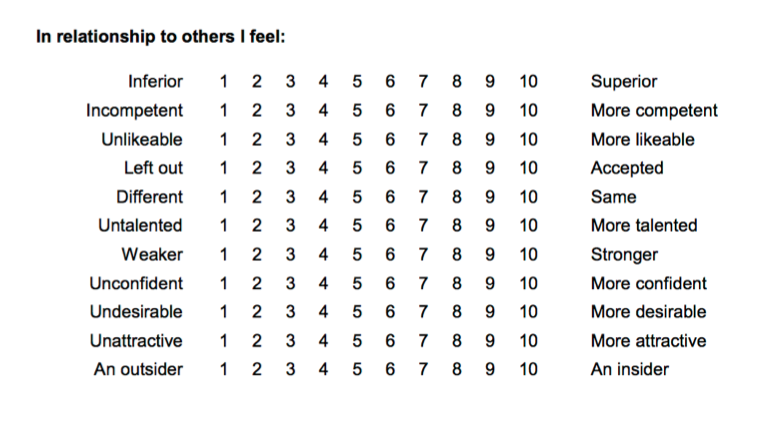
**

*reference:*

Allan, S. & Gilbert, P. (1995). A social comparison scale: Psychometric properties and relationship to psychopathology. Personality and Individual Differences, 19, 293-299.

**Acculturation scale**

A1 How important is it for you to follow Moroccan norms and values?

A2 How important is it for you to have Dutch friends and acquaintances?

| Very important | Important | Neutral | Not important | Totally unimportant |
| --- | --- | --- | --- | --- |

A3 How do you feel inside?

| Mainly Moroccan | Morrocan-Dutch | Mainly Dutch | None of both |
| --- | --- | --- | --- |

A4. With friends, I speak mainly Dutch

A5. With friends, I speak mainly Moroccan

A6. At home, I speak mainly Dutch

A7. At home, I speak mainly Moroccan

A8. I do have many Dutch friends

A9. I do have many Moroccan friends in the Netherlands

A10. I do have many Moroccan friends in Morocco

A11. I celebrate Dutch Holidays

A12. I celebrate Moroccan Holidays

A13. I like Dutch clothing

A14. I like Moroccan clothing

A15. I think it is important to follow Dutch news

A16. I think it is important to follow Moroccan news

| Fully disagree | Disagree | Neutral | Agree | Fully agree |
| --- | --- | --- | --- | --- |

*References:*

- Selten, J. P., & Havenaar, J. M. (2004). *Migratie en Niet-Affectieve Psychose; Add-on study bij protocol “kwetsbaarheid en veerkracht bij niet-affectieve psychose”.*
- Instituut voor Sociale Geneeskunde (2004). *Acculturatieschaal*.

DUDIT - Drug Use Disorders Identification Test


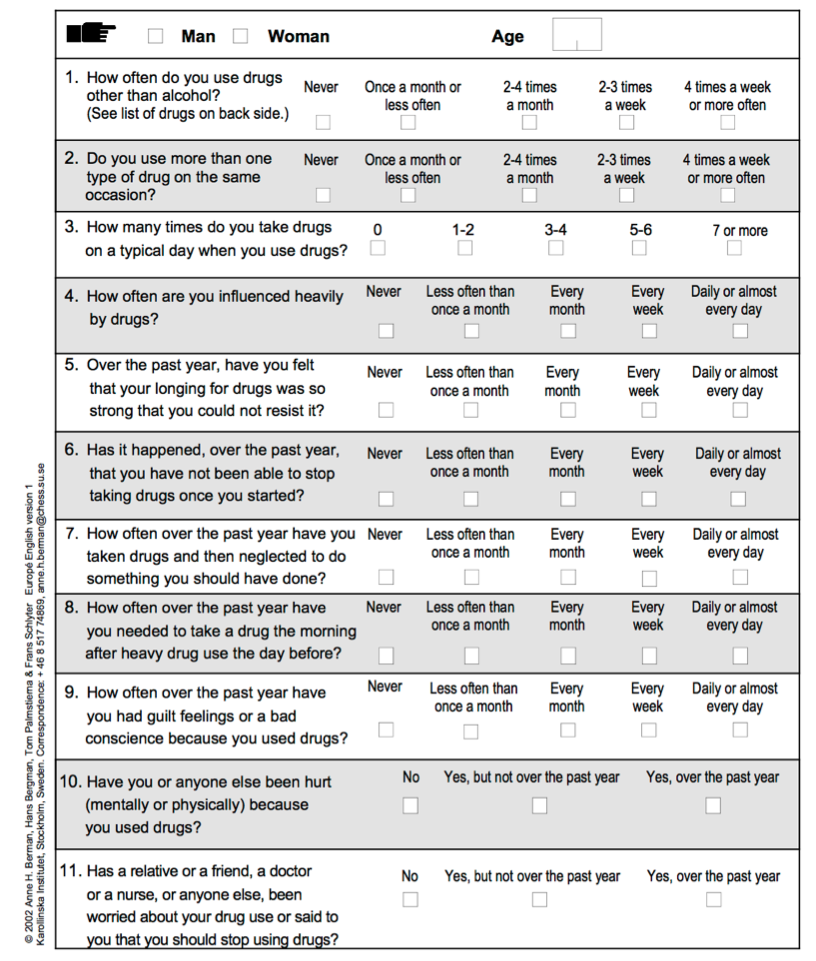


*Reference:*

Selten, J. P., & Havenaar, J. M. (2004). *Migratie en Niet-Affectieve Psychose; Add-on study bij protocol “kwetsbaarheid en veerkracht bij niet-affectieve psychose”.*

voor Sociale Geneeskunde, I. (2004). *Acculturatieschaal*.
